# Fetal heart rate abnormalities during and after external cephalic version: Which fetuses are at risk and how are they delivered?

**DOI:** 10.1186/s12884-017-1547-6

**Published:** 2017-10-17

**Authors:** Simone M. Kuppens, Ida Smailbegovic, Saskia Houterman, Ingrid de Leeuw, Tom H. Hasaart

**Affiliations:** 1 Department of Obstetrics and Gynecology, Catharina Hospital, P.O. Box 1350, 5602 ZA Eindhoven, the Netherlands; 2 Department of Education and Research, Catharina Hospital, P.O. Box 1350, 5602 ZA Eindhoven, the Netherlands

**Keywords:** Pregnancy, Breech presentation, External cephalic version, Fetal heart rate, Fetal distress, Mode of delivery

## Abstract

**Background:**

Fetal heart rate abnormalities (FHR) during and after external cephalic version (ECV) are relatively frequent. They may raise concern about fetal wellbeing. Only occasionally they may lead to an emergency cesarean section.

**Methods:**

Prospective cohort study in 980 women (> 34 weeks gestation) with a singleton fetus in breech presentation. During and after external cephalic version (ECV) FHR abnormalities were recorded. Obstetric variables and delivery outcome were evaluated. Primary outcome was to identify which fetuses are at risk for FHR abnormalities. Secondary outcome was to identify a possible relationship between FHR abnormalities during and after ECV and mode of delivery and fetal distress during subsequent labor.

**Results:**

The overall success rate of ECV was 60% and in 9% of the attempts there was an abnormal FHR pattern. In two cases FHR abnormalities after ECV led to an emergency CS. Estimated fetal weight per 100 g (OR 0.90, CI: 0.87–0.94) and longer duration of the ECV-procedure (OR 1.13, CI: 1.05–1.21) were factors significantly associated with the occurrence of FHR abnormalities. FHR abnormalities were not associated with the mode of delivery or the occurrence of fetal distress during subsequent labor.

**Conclusions:**

FHR abnormalities during and after ECV are more frequent with lower estimated fetal weight and longer duration of the procedure. FHR abnormalities during and after ECV have no consequences for subsequent mode of delivery. They do not predict whether fetal distress will occur during labor.

**Trial registration:**

The Eindhoven Breech Intervention Study, NCT00516555. Date of registration: August 13, 2007.

## Background

External cephalic version (ECV) is the preferred method for reducing breech presentations at labor [1, 2]. Unfortunately, ECV is not without risks, which may lead to concerns about the wellbeing of the unborn child [3–5].

Complications such as vaginal bleeding, placental abruption, preterm rupture of membranes and fetal distress may occur, but they are rare. In less than 4 out of 1000 women this may lead to an emergency cesarean section (CS) [6].

More frequently reported are transient abnormal fetal heart rate (FHR) patterns, which happen in approximately 5% of the cases. These patterns of FHR abnormalities include bradycardias, nonreactive non-stress test, tachycardias and other unspecified patterns [6–10].

Various mechanisms have been proposed to account for these FHR abnormalities such as compression of the umbilical cord or vagal nerve mediated bradycardia (due to direct pressure on the fetus) [8]. Measurement of the umbilical and middle cerebral arterial blood flow support this hypothesis as the application of pressure on the fetal head first decreases blood flow followed by an increase after ECV [11, 12]. These events also may occur during labor.

Another explanation postulated is temporary hypoxia caused by relatively decreased utero-placental blood flow on the basis of increased intrauterine pressure during the procedure [8].

Consequently an increase in cell-free fetal DNA in the maternal circulation was recorded after ECV [13].

Which fetuses are at risk for transient abnormal FHR patterns? And how is the stress of labor tolerated by this group? A study from Lau et al. in 425 women showed that a difficult or successful ECV-procedure and nulliparity are factors predisposing for the occurrence of transient fetal bradycardia [14]. Furthermore, they showed that these fetuses have a higher risk of intrapartum CS because of fetal distress.

We recorded FHR during and after ECV and analyzed the data of almost 1000 women.

Primary outcome was to identify which fetuses are at risk for FHR abnormalities during and after ECV. Secondary outcome was to identify a possible relationship between FHR abnormalities during and after ECV and the occurrence of fetal distress during labor and mode of delivery.

## Methods

### Study design

A prospective observational cohort study was conducted between October 2007 and June 2012 in the Catharina Hospital in Eindhoven, The Netherlands. The study was approved by the Medical Ethical Committee of the Catharina Hospital. Written informed consent was obtained from all participants.

### ECV intervention

The Obstetric Department of the Catharina Hospital has extensive experience in ECV. All ECV procedures during the study period were performed by the same four trained operators of whom two obstetricians and two midwifes. Since we prefer a filled bladder, women with an empty bladder were advised to drink before the procedure.

The hands of one staff member concentrated on the breech, while the hands of the other staff member concentrated on the fetal head. In order to prevent excessive pressure on the fetus, the manipulation between the pair of hands was rather consecutive than simultaneous. ‘Forward somersault’ was the preferred method to achieve cephalic position, and a ‘backward flip’ was an alternative strategy for nulliparous women with a frank breech presentation [15].

Before ECV, ultrasound was used to determine fetal position, estimated fetal weight (EFW), placental localization and amniotic fluid index (AFI). A tocolytic agent (Atosiban, 6.75 mg intravenously) was used in all ECV attempts.

Before and after each ECV procedure the fetal heart rate was monitored by cardiotocography (CTG). During the ECV procedure itself an assistant was monitoring the FHR by ongoing ultrasound.

### Participants

Pregnant women who underwent ECV for breech presentation were included. Exclusion criteria were maternal age under 18 years, gestational age less than 34 weeks, a history of CS, no mastery of the Dutch language and contraindications for ECV.

### Assessments

Before ECV, several obstetric factors were documented by the operators: maternal age (years), parity (primi- or multiparous), gestational age at ECV (weeks and days), type of breech (frank versus non-frank), placental localization (anterior versus non-anterior), AFI (≤10 or >10 cm), engagement of the fetal breech (above or in pelvic inlet), palpability of the fetal head (yes or no), fundal height (in cm) and EFW by ultrasound (in gram). Engagement of the fetal breech was a subjective assessment measured by the obstetricians and the midwife who performed the ECV.

During ECV, ultrasound was used to monitor fetal position and FHR. Episodes of fetal bradycardia were recorded. In all patients the number and total duration of the ECV attempts were recorded (in minutes). After ECV, the FHR was recorded by CTG for at least 60 min and abnormalities such as decelerations, bradycardia (as a FHR below 110 bpm) or tachycardia (as FHR above 170 bpm) were registered.

After delivery, characteristics of the delivery in cephalic presenting labors were recorded (spontaneous vaginal delivery, assisted vaginal delivery or CS), fetal gender (male or female), birth weight (in grams) and the presence of fetal distress during labor (yes or no).

Primary outcome was to identify which fetuses are at risk for FHR abnormalities during and after ECV. Secondary outcome was to identify a possible relationship between FHR abnormalities during and after ECV and the occurrence of fetal distress during subsequent labor.

### Data analysis and processing

The mean and standard deviation, median and range or numbers of patients were estimated for each baseline characteristic. All characteristics were shown separately for cases with or without FHR abnormalities. Differences between groups for normally distributed variables were estimated with a t-test and for skewed variables with a Mann-Whitney test. Univariate logistic regression was used to select variables significantly associated with FHR abnormalities (dependent variable; odds ratio (OR) and 95% confidence interval (CI)). Subsequently, we evaluated the significant variables (*P* < 0.05) from the univariate analysis in a multivariate logistic regression model.

A *p*-value <0.05 was considered statistically significant. Because fundal height, EFW and birth weight are highly correlated, only EFW was put into the multivariate logistic regression model.

Finally, a possible relation between FHR abnormalities (during and after ECV) with fetal distress and mode of delivery was studied with a Chi-square test. Statistical analysis was carried out using the Statistical Package for Social Sciences for Windows 23.0 (SPSS).

## Results

In total 980 ECV procedures were analyzed (Table 1) and in almost two thirds of the cases, these were primiparous women, *n* = 626 (64%).

**Table 1:** Baseline characteristics of 980 patients who underwent ECV between October 2007 and June 2012

	Total (*n* = 980)	CTG-abnormalities during or after ECV (*n* = 86)	No CTG-abnormalities during or after ECV (*n* = 894)	*P*-value
Maternal age (years)				
Mean, standard deviation	31.2 (4.1)	31.2 (3.7)	31.3 (4.2)	0.9
Range	17–46	21–40	17–46	
Parity				
Primiparous	626 (64%)	59 (69%)	567 (63%)	0.3
Multiparous	354 (36%)	27 (31%)	327 (37%)	
Gestational age at ECV (weeks and days)				
Median	35 + 6	36 + 0	35 + 6	0.4
Range	34 + 0–41 + 5	34 + 0–38 + 0	34 + 2–41 + 5	
Type of breech				
Non-Frank	323 (34%)	26 (30%)	297 (34%)	0.5
Frank	630 (66%)	60 (70%)	570 (66%)	
Estimated Fetal Weight (EFW in grams)				
Mean, standard deviation	2591 (316)	2505 (276)	2599 (319)	0.009
Range	1700–4000	1880–3526	1700–4000	
Fundal height (centimeters)				
Mean, standard deviation	32.8 (1.7)	32.2 (1.2)	32.8 (1.7)	0.01
Range	26–40	29–35	26–40	
Engagement				
Breech above pelvic inlet	481 (50%)	35 (41%)	446 (51%)	0.08
Breech in pelvic inlet	485 (50%)	51 (59%)	434 (49%)	
Head palpable				
No	64 (7%)	4 (5%)	60 (7%)	0.5
Yes	912 (93%)	82 (95%)	830 (93%)	
Placenta location				
Posterior/lateral	582 (60%)	55 (65%)	527 (59%)	0.3
Anterior	394 (40%)	30 (35%)	364 (41%)	
Amniotic Fluid Index (AFI in cm)				
> 10	392 (40%)	25 (29%)	367 (41%)	0.03
≤ 10	586 (60%)	61 (71%)	525 (59%)	
Duration ECV (in minutes)				
Median	2.50	4.15	2.45	< 0.001
Range	0.15–16.05	0.30–16.05	0.15–15.45	
Number of ECV attempts				
Mean, standard deviation	2.2 (1.4)	2.3 (1.4)	2.2 (1.4)	0.5
Range	1–7	1–6	1–7	
ECV success				
Yes	584 (60%)	55 (64%)	529 (59%)	0.4
No	396 (40%)	31 (36%)	365 (41%)	
Delivery mode				
Spontaneous vaginal	494 (50%)	48 (56%)	446 (50%)	0.3
Assisted vaginal and caesarean section	486 (50%)	38 (44%)	448 (50%)	
Gender newborn				
Boy	440 (45%)	40 (48%)	400 (45%)	0.6
Girl	537 (55%)	44 (52%)	493 (55%)	
Birth weight (grams)				
Median	3322	3201	3325	0.009
Range	1700–4705	1782–4280	1700–4705	

Mean gestational age at the moment of the ECV was 35 weeks +6 days (range from 34 weeks to 41 weeks and 5 days). More fetuses were in frank breech position (*n* = 630, 66%) than in non-frank (*n* = 323, 34%). In half of the cases, the breech was in the pelvic inlet. Median duration of the total ECV was 2.50 min (range from 0.15 to 16.05 min), with a mean number of 2.2 (± 1.4) attempts per procedure.

ECV was successful in 584 (60%) of the procedures. In 86 (9%) of the procedures a FHR abnormality occurred during the ECV.

Lower estimated fetal weight on ultrasound (mean 2505 versus 2599 g; *p* = 0.009), less fundal height (mean 32.2 versus 32.8 cm; *p* = 0.01), longer duration of the ECV procedure (median 4.15 versus 2.45 min; *p* < 0.001), lower AFI ≤ 10 cm (29% versus 41%; *p* = 0.03) and lower neonatal birth weight (mean 3201 versus 3325 g; p = 0.009) were risk factors significantly associated with the occurrence of FHR abnormalities.

Multivariate analysis (Table 2) shows that FHR abnormalities were associated with longer duration of ECV (OR 1.13, CI: 1.05–1.21; *p* < 0.001) and with lower EFW per 100 g (OR 0.90, CI: 0.87–0.94; *p* = 0.01).

**Table 2:** Odds ratios (OR) for fetal heart rate abnormalities during and after ECV

	Univariate		Multivariate	
Variable	O.R.	95% C.I.	O.R.	95% C.I.
Maternal age (years)	1.00	0.94–1.05		
Gestational age at ECV (weeks and days)	1.02	0.80–1.30		
Nulliparity	0.79	0.49–1.28		
Engagement of breech	1.50	0.96–2.35		
Frank breech	1.20	0.74–1.95		
Amniotic fluid index ≤ 10 cm	1.71	1.05–2.77	1.57	0.95–2.58
Placenta anterior	0.79	0.50–1.26		
Duration of ECV (minutes)	1.14	1.07–1.22	1.13	1.05–1.21
ECV success	0.82	0.52–1.29		
Estimated fetal weight (per 100 g)	0.90	0.87–0.94	0.90	0.87–0.94

In two cases FHR abnormalities after ECV led to an emergency CS.

The first case was a 31-year old multiparous woman, G4P2 with gestational age of 35 weeks +5 days. She had two previous vaginal deliveries and one spontaneous abortion. After a successful ECV procedure there was a fetal bradycardia (90 beats per minute) during 10 min followed by tachycardia and decelerations. An emergency cesarean section was performed and after 25 min a female fetus, 2230 g in cephalic position was born with Apgar scores 9 after 1 min, 10 after 5 min and normal umbilical cord blood gas analysis. The second case was a 32-year old primiparous woman with gestational age 37 weeks. After a non-successful ECV procedure there was a fetal bradycardia of 80 beats per minute. No clinical signs of placental abruption were present. An emergency CS was performed and after 20 min a male fetus with a weight of 2540 g was born with low Apgar scores of 3 after 1 min and 8 after 5 min. Umbilical artery blood gas analysis showed a pH of 6.92 with base excess of −7/9 mmol/l. Neonatal recovery was uneventful. In both cases no explanation for the fetal distress was found (no signs of placental abruption or fetal maternal hemorrhage). Both cases were excluded from analysis.

**Fig. 1:**
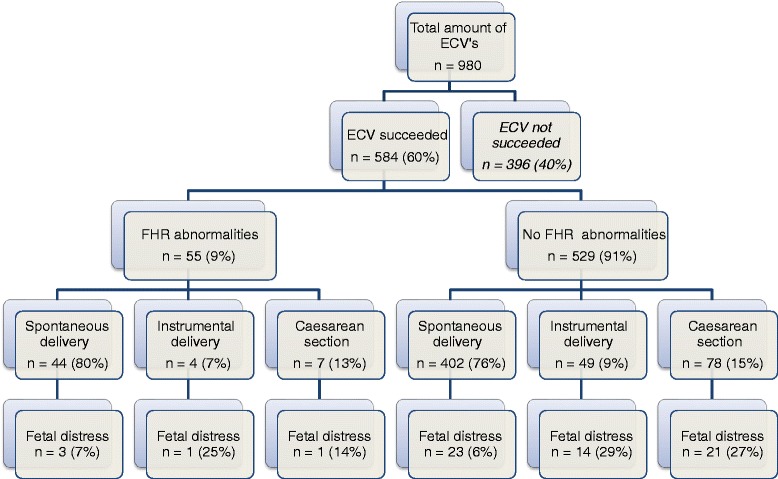
Labor outcome after successful ECV in 584 patients and the occurrence of fetal distress

FHR abnormalities during and after successful ECV occurred in 9% of women (*n* = 55). As illustrated in Fig. 1, mode of delivery (spontaneous, instrumental or CS) was similar between women with or without FHR during and after successful ECV. Moreover, the occurrence of fetal distress during subsequent labor between these two groups was also similar.

## Discussion

Factors associated with FHR abnormalities during and after ECV are estimated fetal weight (EFW) and the duration of the ECV-attempt.

FHR abnormalities during and after ECV are not associated with fetal distress during labor or mode of delivery.

In our study 9% of the ECV’s were complicated by FHR abnormalities. In the literature abnormal CTG findings are reported up to 5.7% [7]. Because the FHR was checked both during and after ECV, we found a higher number (9%) of FHR abnormalities.

Various mechanisms have been proposed to account for FHR abnormalities such as temporary hypoxemia caused by decreased utero-placental bloodflow [8]. Since placental reserve is less in small for gestational age fetuses, they may be more prone to FHR abnormalities because of hypoxemia. In the literature this is confirmed by measurements of the pre-ECV mean cerebral artery pulsatility index which shows an association between a non-reassuring fetal status after ECV and brain-sparing effects in the mildly compromised growth retarded fetus [16].

Duration of the ECV was also associated with FHR abnormalities during and after ECV. Because longer duration of the ECV may eventually cause disturbances at the uteroplacental unit, this may lead to FHR abnormalities after ECV [17].

Furthermore, direct pressure on the fetus can also cause vagal nerve compression and hence lead to FHR abnormalities [8]. Since we did not measure exerted force during ECV, we could not account for this variable. Studies in which the exerted force during ECV was measured using pressure-sensing gloves show that the greater the force applied, the more severe the reduction of blood flow in middle cerebral artery and umbilical artery. But no correlation was found between FHR and the applied force [12, 17]. Nevertheless, duration as well as force used during ECV should be limited.

Since more pressure is needed when there is a more intense uterine and abdominal muscle tone or engagement of the breech, ECV was accompanied with more FHR abnormalities in nulliparous women in the study by Lau et al. [14]. However, we could not confirm this finding.

It has been postulated that FHR abnormalities during ECV could imply that the fetus is more prone to stress during labor. However, in our study the risk of fetal distress during labor was the same in the groups with or without FHR abnormalities during and after successful ECV. Moreover, their mothers were not at risk for CS and instrumental vaginal delivery. This is in contradiction with a study from Leung who demonstrated that fetuses presenting with transient bradycardia post-ECV had a two-fold increased risk of intrapartum CS for non-reassuring fetal status.

A major strength of the current study is its large sample size and the fact that ECV was performed in one obstetric department by a small team of trained experts following the same protocol. The ratio between nulliparous and parous women at the ECV outpatient clinic in this study is in line with the incidence in the general population suggesting that the sample is representative with regard to an important determinant of ECV outcome: parity.

Furthermore, all data were prospectively recorded. Recording of the duration of the ECV was carefully obtained under supervision of an unbiased research nurse who accompanied all ECV procedures.

FHR abnormalities during and after ECV are relatively frequent (9%). In 2 cases (0.2%) ECV led to FHR abnormalities requiring emergency CS. This is in line with the literature [6, 7].

In our opinion, this small risk of emergency CS doesn’t justify a policy of overnight fasting. Therefore, we advise women with an empty bladder to drink before the procedure. This helps to lift the breech out of the pelvic inlet.

FHR changes following ECV appear to represent only a transient response to the procedure [9]. However duration as well as force of manipulation should be limited. Bradycardia up to 5 min is regarded as not harmful. [14] In the event of fetal bradycardia, the ECV should be discontinued until a normal pattern returns.

## Conclusion

FHR abnormalities during and after ECV are more frequent with lower estimated fetal weight and longer duration of the procedure. FHR abnormalities during and after ECV have no consequences for subsequent mode of delivery. They do not predict whether fetal distress will occur during labor.

## Abbreviations

AFI: Amniotic fluid index
BPM: Beats per minute
cm: Centimeters
CS: Cesarean section
CTG: Cardiotocography
ECV: External cephalic version
EFW: Estimated fetal weight
FHR: Fetal heart rate
